# Endothelial Dysfunction Markers in Ovarian Cancer: VTE Risk and Tumour Prognostic Outcomes

**DOI:** 10.3390/life14121630

**Published:** 2024-12-09

**Authors:** Inês Guerra de Melo, Valéria Tavares, Joana Savva-Bordalo, Mariana Rei, Joana Liz-Pimenta, Deolinda Pereira, Rui Medeiros

**Affiliations:** 1 Molecular Oncology and Viral Pathology Group, Research Centre of IPO Porto (CI-IPOP), Pathology and Laboratory Medicine Department, Clinical Pathology SV/RISE@CI-IPOP (Health Research Network), Portuguese Oncology Institute of Porto (IPO Porto)/Porto Comprehensive Cancer Centre (Porto. CCC), 4200-072 Porto, Portugal; ines.melo@ipoporto.min-saude.pt (I.G.d.M.); valeria.tavares@ipoporto.min-saude.pt (V.T.); 2 Faculty of Medicine, University of Porto (FMUP), 4200-072 Porto, Portugal; joanalizpimenta@gmail.com; 3 ICBAS—Instituto de Ciências Biomédicas Abel Salazar, Universidade do Porto, 4050-313 Porto, Portugal; 4 Department of Medical Oncology, Portuguese Institute of Oncology of Porto (IPO Porto), 4200-072 Porto, Portugal; joana.sa@ipoporto.min-saude.pt (J.S.-B.); dpereira@ipoporto.min-saude.pt (D.P.); 5 Department of Gynaecology, Portuguese Institute of Oncology of Porto (IPO Porto), 4200-072 Porto, Portugal; marianacruzrei@gmail.com; 6 Department of Medical Oncology, Centro Hospitalar de Trás-os-Montes e Alto Douro (CHTMAD), 5000-508 Vila Real, Portugal; 7 Faculty of Health Sciences, Fernando Pessoa University, 4200-150 Porto, Portugal; 8 Research Department, Portuguese League Against Cancer (NRNorte), 4200-172 Porto, Portugal

**Keywords:** ovarian neoplasms, venous thrombosis, polymorphism, endothelium, nitric oxide, von Willebrand factor, P-selectin, prognosis

## Abstract

Ovarian cancer (OC) presents daunting lethality rates worldwide, with frequent late-stage diagnosis and chemoresistance, highlighting the need for improved prognostic approaches. Venous thromboembolism (VTE), a major cancer mortality factor, is partially driven by endothelial dysfunction (ED). ED’s pro-inflammatory state fosters tumour progression, suggesting a VTE-independent link between ED and cancer. Given this triad’s interplay, ED markers may influence OC behaviour and patients’ prognosis. Thus, the impact of ED-related genes and single-nucleotide polymorphisms (SNPs) on OC-related VTE and patient thrombogenesis-independent prognosis was investigated. *NOS3* upregulation was linked to lower VTE incidence (χ^2^, *p* = 0.013), while *SELP* upregulation was associated with shorter overall survival (log-rank test, *p* = 0.048). Dismissing patients with VTE before OC diagnosis, *SELP* rs6136 T allele carriers presented lower progression-free survival (log-rank test, *p* = 0.038). Nevertheless, due to the SNP minor allele underrepresentation, further investigation is required. Taken together, ED markers seem to exhibit roles that depend on the clinical context, such as tumour-related thrombogenesis or cancer prognosis. Validation with larger cohorts and more in-depth functional studies are needed for data clarification and potential therapeutic strategies exploitation to tackle cancer progression and thrombosis in OC patients.

## 1. Introduction

Tumour heterogeneity and therapy resistance remain the major obstacles to effective treatment of malignant diseases [1]. This is particularly evident in ovarian cancer (OC), displaying five-year survival rates below 50% in most countries, particularly when considering advanced stages [2,3]. Indeed, late-stage diagnosis combined with the common acquisition of chemoresistance makes OC the most lethal gynaecological tumour on a global scale [2,4,5,6]. The histological subtype primarily accountable for gynaecological cancer-related deaths is serous carcinoma (SC), particularly high-grade tumours [7,8].

Beyond the International Federation of Gynaecology and Obstetrics (FIGO) staging, histological and grade classification, prognosis assessment also relies on baseline serum cancer antigen 125 (CA-125) levels, the extent of debulking surgery and chemotherapy regimens, as independent key factors [2,9]. However, these prognostic determinants alone are not sufficient to fully capture the complexity of the disease [10,11]. Thus, more reliable biomarkers are crucial for improving prognostic accuracy, treatment strategies and disease monitoring, enabling a more personalised and effective OC management.

Venous thromboembolism (VTE), including deep vein thrombosis (DVT) and pulmonary embolism (PE), is a common cardiovascular complication among patients with cancer, also representing their second leading cause of death [6,12,13]. These patients face a fourfold to ninefold heightened vulnerability to venous thrombogenesis compared with the general population, which can be explained by factors related to the patient (e.g., patient’s advanced age), tumour (e.g., cancer primary site) and antineoplastic treatments (e.g., chemotherapy) [13,14,15,16]. Among OC patients, beyond patient and cancer-related factors, platinum-based chemotherapy, antiangiogenic treatment, and pelvic surgery, significantly increase the incidence of VTE, ranking this malignancy among those with the highest thrombotic risk [17,18]. Indeed, the connection between cancer pathophysiology and VTE has garnered significant attention. In a bidirectional relationship, malignancy and cancer-associated thrombosis (CAT) serve as a mutual risk factor for each other and exert a significant impact on each other’s mortality rates [19,20]. Given the negative effect of CAT, a better comprehension of its pathogenesis is crucial.

Whether in the general population or in cancer patients, VTE development can be elucidated by the Virchow Triad, which encompasses three key factors: stasis of blood flow, blood hypercoagulability and endothelial dysfunction (ED). The latter, defined as an alteration in the normal function of the endothelial cells (ECs) lining the interior of blood vessels, is pivotal in VTE onset [15,21]. The core of ED is an imbalance between endothelium-derived relaxing factors (EDRFs) and constricting factors (EDCFs). Among various EDRFs, nitric oxide (NO) plays the most critical role in ED [21,22]. In addition to endothelial permeability, decreased NO bioavailability induces the expression of important cell adhesion molecules (CAMs), namely P-selectin, E-selectin, von Willebrand factor (vWF), intercellular adhesion molecule 1 (ICAM-1) and vascular cell adhesion molecule-1 (VCAM-1), which facilitate cell-to-cell interaction, promoting the migration and adhesion of leucocytes [23]. Also, the TGF-β co-receptor endoglin (ENG) is upregulated, further contributing to a pro-inflammatory state that precedes VTE [24,25,26,27].

Beyond VTE, ED is a critical factor in the pathogenesis of a vast spectrum of other metabolic and cardiovascular diseases (CVD) [22,24,28]. A relevant bridge to cancer is also formed as the pro-inflammatory state of ED promotes tumour growth and progression. Additionally, the inhibition of vasodilation supports cell proliferation and anti-apoptotic responses, reinforcing the association between ED and cancer [29,30]. Collectively, the triad formed by ED, VTE and cancer could open potential avenues for OC management, particularly from a genetic perspective. Indeed, ED is thought to have a genetic basis, with single-nucleotide polymorphisms (SNPs) being particularly valuable [31,32]. Given their frequency and relevance at a population level, investigating SNPs implicated in ED susceptibility may support CAT’s prediction and early diagnosis, improve OC prognosis assessments and enable more personalised and targeted tumour treatments. In this context, this study aimed to explore the implications of ED biomarkers on the occurrence of OC-related VTE and the prognosis of OC patients, independent of thrombosis.

## 2. Materials and Methods

### 2.1. Population Description

A retrospective hospital-based cohort study was carried out enrolling epithelial OC (EOC) patients of European ancestry admitted for first-line treatment from March 2017 to December 2023 at the Department of Gynaecology and Oncology of IPO Porto. Patients were excluded when underage, only admitted for a second opinion or with follow-up completed at other institutions. Ultimately, 98 EOC patients (cohort A) with available biological material were recruited. All participants signed a consent form according to the principles of the Helsinki Declaration before their enrolment in the research.

All EOC cases were staged using the FIGO staging system [33]. Adding to this, the Response Evaluation Criteria in Solid Tumours (RECIST) version 1.1 was considered when evaluating the tumour response to chemotherapy [34]. Follow-up records, as well as demographic and clinicopathological information, were retrieved by consulting all patients’ medical data files.

In summary, the mean age of the patients was 63.2 years, and most of them were post-menopausal (*n* = 79, 80.6%) and had received their diagnosis at advanced cancer stages (FIGO III/IV; *n* = 74, 75.5%). A total of 82 (83.7%) patients were diagnosed with serous tumours. Most (*n* = 41, 41.8%) received standard treatment (cytoreductive surgery followed by chemotherapy with carboplatin/cisplatin combined with paclitaxel). Complete or optimal surgical resection was achieved for 45 (45.9%) patients. The median follow-up time in the study was 25.5 months.

CAT was defined as a VTE event occurring six months before to two years after OC detection [35]. Therefore, analyses involving VTE included only patients with a minimum follow-up of two years, with those who died during this period also considered. Notably, active screening is not an established clinical protocol at IPO Porto and thus it was not performed. Among the 80 OC patients with sufficient follow-up time, 17 (21.3%) developed OC-related VTE, with 6 cases occurring before and 11 after OC diagnosis. The median time interval between VTE and OC diagnosis was 3.5 months for thrombotic cases preceding OC diagnosis and 9.0 months for post-diagnosis cases. Regarding the type of VTE, ten events (58.8%) were DVT, five (29.4%) were PE, one involved both DVT and PE and one occurred in an unusual location.

This study had previously received approval from the ethics committee at IPO Porto (CES IPO: 69/021; 11 March 2021).

### 2.2. Sample Collection, Genomic DNA and RNA Extraction

Using a standard phlebotomy approach, venous peripheral blood samples of patients were collected into EDTA collection tubes prior to their first-line chemotherapy.

Following the manufacturer’s instructions, the QIAamp DNA Blood Mini Kit (Cat. No. 51106, Qiagen, Hilden, Germany) was used to isolate genomic deoxyribonucleic acid (DNA) from the blood samples of each patient for SNP genotyping.

The isolation of peripheral blood components (PBCs) was conducted as described elsewhere [10]. Total ribonucleic acid (RNA) for messenger RNA (mRNA) analyses was extracted from the cellular fraction of the peripheral blood samples using the GRS RNA kit—Blood & cultured cells (#GK08.0100, Grisp Research Resolutions^®^, Porto, Portugal) as per the manufacturer’s instructions.

The concentration and purity of the DNA and RNA samples were verified using the NanoDrop Lite spectrophotometer by Thermo Fisher Scientific^®^ (Waltham, MA, USA). Only RNA samples with A260/280 ≥ 1.90 were admitted. Once extracted and quantified, all DNA samples were stored at −20 °C, while the RNA samples were stored at −80 °C until use.

### 2.3. Polymorphism Selection and Genotyping

Several criteria were used to guide the identification of relevant SNPs associated with ED. The genetic variants were selected if they: (1) were located within genes implicated in ED, (2) had a demonstrated impact on the expression or activity of the respective encoded proteins, (3) had a role in CVD and metabolic diseases and/or cancer and (4) had available TaqMan genotyping assays. Finally, SNPs related to the same gene and in strong linkage disequilibrium (LD) (meaning with a r^2^ value higher than 0.9) were dismissed. Applying these criteria, three SNPs were selected: *NOS3* rs2070744, *SELP* rs6136 and *VWF* rs1063856.

SNP genotyping was carried out in a StepOne Plus quantitative real-time (qRT)-PCR system (Applied Biosystems^®^, Foster City, CA, USA), leveraging the TaqMan^®^ Allelic Discrimination approach. Thermal cycles for DNA amplification were the following: activation of Taq DNA-polymerase (10 min at 95 °C); denaturation of DNA chains (15 s of 45 cycles at 95 °C) and primer pairing and extension (1 min at 60 °C). Each reaction was conducted with a 6.0 µL mix, including 2.5 µL of TaqPath^TM^ ProAmp^TM^ Master Mix (1×), 2.375 µL of sterile water, 0.125 µL of TaqMan^®^ Genotyping Assay (C__15903863_10 for *NOS3* rs2070744, C__11975277_20 for *SELP* rs6136 and C___3288406_30 for *VWF* rs1063856), and 1.0 µL of genomic DNA, incorporating negative controls (without genetic material) to assess false positives. A double-sampling strategy of at least 10.0% randomly selected samples was performed to ensure SNP genotyping quality. StepOne Software (version 2.3 Applied Biosystems^®^, Foster City, CA, USA) was employed to analyse the data on DNA amplification data. The genotyping results were evaluated by two researchers with no previous knowledge regarding the demographic and clinicopathological details of patients involved in the study.

### 2.4. Gene Selection

A comprehensive literature review on the molecular profile associated with ED was recently conducted by our research group [36]. Based on this review, genes were selected by prioritising those expressed by PBCs, particularly monocytes, neutrophils, and platelets. Furthermore, different phases of ED—immediate endothelial activation, delayed endothelial activation, and established ED—were considered, leading to the selection of the following genes: *NOS3*, *SELP*, *ICAM1*, *ENG*, and ET-1 encoding gene (*EDN1*).

Cohort A was subsampled by filtering out patients who: (1) had a history of other malignancies; (2) were breastfeeding or pregnant at the time of diagnosis; (3) had a history of autoimmune diseases or were undergoing immunosuppressive therapies; (4) had acute infections at cancer diagnosis; (5) were undergoing anticoagulant treatments for diseases other than VTE; and (6) possessed the polymorphisms Factor V Leiden (*F5* rs6025) and *F2* (Factor II encoding gene) rs1799963. The resultant cohort—cohort B—was comprised of 55 OC patients, who were subject to gene expression analysis.

### 2.5. cDNA Conversion and Gene-Relative Quantification

Complementary DNA (cDNA) strands were generated from total RNA samples by using the High-Capacity cDNA Reverse Transcription Kit (Applied Biosystems^®^, Carlsbad, CA, USA) as per the manufacturer’s instructions, and a MycyclerTM Thermal cycler (Bio-Rad Laboratories, Hercules, CA, USA) with the recommended cycle conditions (10 min at 25 °C, 120 min at 37 °C, and 5 min at 85 °C). All reactions incorporated negative controls.

The relative quantification of gene expression was performed with the StepOne Plus qRT-PCR system. Each reaction was executed using a 10.0 µL mixture containing: 5.0 µL of 2× TaqMan^TM^ Gene Expression Master Mix and 0.5 µL of TaqMan^TM^ 20× Gene Expression Assays, both by Applied Biosystems^®^ (Foster City, CA, USA); and 3.0 µL of nuclease-free water and 1.5 µL of cDNA sample. The selected assays were the following: Hs01574665_m1 (*NOS3*), Hs00923996_m1 (*ENG*), Hs00174961_m1 (*EDN1*), Hs00164932_m1 (*ICAM1*), and Hs00927900_m1 (*SELP*). Glyceraldehyde-3-phosphatedehydrogenase (*GAPDH*) and hypoxanthine phosphoribosyl transferase 1 (*HPRT1*) were tested as endogenous controls with the assays Hs03929097_g1 and Hs02800695_m1, respectively. All reactions followed the same thermal cycling conditions: 50 °C for 2 min, 95 °C for 10 min followed by 45 cycles of 15 s at the same temperature, and 1 min at 60 °C. Negative controls (without cDNA) were included in all reactions to assess false positives, and a triple-sampling strategy of all samples was employed. The amplification of all targets and endogenous controls of each sample was conducted on the same plate. Reactions with standard deviation (SD) values of cycle thresholds (Ct) >0.5 were excluded. Ct values were generated with the same set baseline and threshold values for each plate by using the qRT-PCR analysis software from the Thermo Fisher Connect platform (Thermo Fisher Scientific, Waltham, MA, USA).

### 2.6. Statistical Analysis

Data analysis was conducted using IBM SPSS Statistics for Windows (version 29, IBM Corp., Armonk, NY, USA). The assessment of data distribution relied on the Kolmogorov-Smirnov test. Continuous variables were dichotomised using the mean value as the cut-off for normally distributed data or the median for non-normally distributed data.

The genotype distribution of each SNP in this study was compared with that of the Iberian population (https://www.ensembl.org/index.html, accessed on 18 August 2024). The Hardy−Weinberg equilibrium (HWE) was tested using the chi-square test (χ^2^). Associations of the SNPs with patients’ clinical and demographic characteristics, including CAT status, as well as the expression of ED-related genes, were analysed using χ^2^.

The gene-normalised relative expression was computed using the Livak method with *GAPDH* as the endogenous control due to its stable expression when compared to *HPRT* (i.e., lower SD values). By applying the interquartile range (IQR), the most severe outliers in the levels of normalised relative expression of each gene were identified and further dismissed. Furthermore, four expression profiles categorising gene-normalised relative expression were defined: (A) low vs. high considering median value expression; (B) low vs. intermediate vs. high expression values considering the terciles; (C) low (first and second terciles) vs. high (third tercile) expression; and (D) low (first tercile) vs. high (remaining distribution) by combining the terciles.

Depending on the data distribution, the Mann–Whitney *U* test or Student-*t* test was used to evaluate the statistical differences in the gene-normalised relative expression levels according to VTE status (VTE-negative/VTE-positive). When examining these associations considering patients without VTE, those with the condition before OC diagnosis, and those after, the Kruskal−Wallis test or one-way analysis of variance followed by Dunnett’s test was employed. χ^2^ was further used for confirmation. The same test was employed to assess the associations of gene expression levels with ED-related SNPs and patients’ clinical and demographic characteristics.

The impact of ED-related markers on the patient’s progression-free survival (PFS) and overall survival (OS) was evaluated. PFS was defined as the interval between the initiation of cancer treatment and either the date of recurrence, progression, related mortality, or the patient’s last clinical evaluation. OS was deemed the time from the patient’s diagnosis until death (all causes included) or the last clinical evaluation. Survival curves were generated with the Kaplan−Meier method, while the survival probabilities were examined using the log-rank test. The most fitting genetic model for each variant (recessive or dominant) was established after analysing the survival curves under the additive genetic model. The risks of tumour progression and patient death were computed employing the Cox proportional hazards model.

All tests conducted were two-sided and a 5.0% significance level was established. Furthermore, *p*-values between 0.050 and 0.060 were deemed marginally significant.

## 3. Results

### 3.1. Distribution of SNP Genotypes

The distribution of the variants’ genotypes is represented in Table 1. The ED-related SNPs were in HWE (χ^2^, *p* > 0.050), demonstrating no significant deviation from expected genotype frequencies.

### 3.2. ED Markers and Clinical Characteristics of OC Patients

While no significant associations were found for the evaluated ED-related genes [regardless of the expression profile (χ^2^, *p* > 0.050)], the SNPs were associated with several demographic and clinicopathological features of the patients (Table 2). The *NOS3* rs2070744 CC genotype was more prevalent among patients with a CVD and/or metabolic disease history (CC vs. CT vs. TT; χ^2^, *p* = 0.049). This association was corroborated when considering patients with both conditions (CC vs. CT/TT; χ^2^, *p* = 0.023). Furthermore, the SNP TT genotype was more frequently associated with using poly (ADP-ribose) polymerase (PARP) inhibitors (PARPi) in both the additive (CC vs. CT vs. TT; χ^2^, *p* = 0.029) and dominant (CC/CT vs. TT; χ^2^, *p* = 0.025) genetic models. A marginal association was observed between this SNP and international normalised ratio (INR) baseline levels (CC/CT vs. TT; χ^2^, *p* = 0.059).

The *SELP* rs6136 G allele was more frequently associated with a CVD and/or metabolic disease history (GG/GT vs. TT; χ^2^, *p* = 0.018). Conversely, the T allele appeared to be more prevalent in patients at advanced FIGO stages (III/IV; χ^2^, *p* = 0.042). However, the findings should be evaluated carefully given the underrepresentation of the GG genotype, reflecting its low minor allele frequency (MAF) (8.9%) in the Iberian population.

The *VWF* rs1063856 TT genotype was strongly associated with a history of arterial thromboembolism (ATE) in both the dominant (CC/CT vs. TT; χ^2^, *p* = 0.018) and additive (CC vs. CT vs. TT; χ^2^, *p* = 0.021) models. Additionally, an association between this genotype and anticoagulant use before OC diagnosis yielded a marginal result (χ^2^, *p* = 0.054).

### 3.3. ED-Markers and VTE Status

Two different approaches were used to assess this association. The first approach grouped patients into two categories—those with (VTE positive) and those without VTE (VTE-free/negative). In the second approach, VTE-free patients were compared to those with the condition before OC diagnosis and those presenting VTE after tumour diagnosis. Although no significant associations were detected for the SNPs, [regardless of the approach used (χ^2^, *p* > 0.050)], in the first approach, *NOS3* was marginally associated with VTE status using the Mann–Whitney *U* test on cohort B. Namely, patients without VTE were found to present higher gene expression levels compared to those with the condition (*p* = 0.057). Regarding χ^2^ for confirmation, *NOS3* expression levels were once again found to be significantly associated with VTE risk in both approaches (Table 3). In the first approach, higher *NOS3* expression was associated with a lower risk of VTE (profile B; χ^2^, *p* = 0.013). Notably, VTE events were only reported in the low and intermediate *NOS3* expression groups. In the second approach, VTE events were only observed in the low and intermediate expression groups, with none occurring in the high expression group (profile B; χ^2^, *p* = 0.027). The χ^2^ did not reveal any additional significant association between the expression of the ED-related genes and VTE status (*p* > 0.050).

### 3.4. SNPs’ Impact on Clinical Outcome of OC Patients (Independently of VTE)

**Figure 1:**
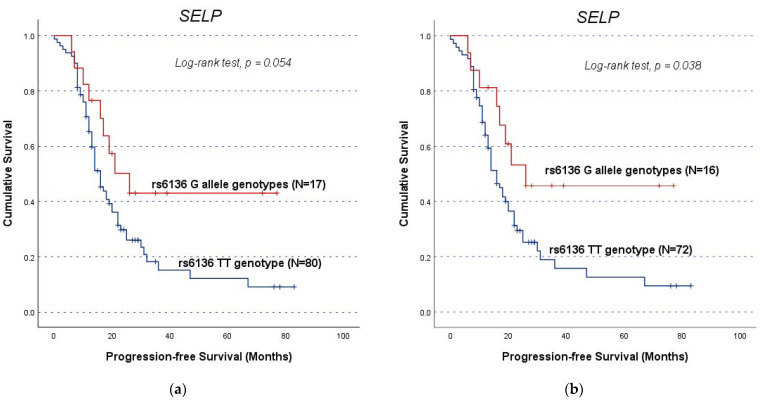
Progression-free survival (PFS) by Kaplan−Meier and log-rank test for OC patients, according to *SELP* rs6136 genotype distribution. The TT genotype carriers had lower survival times: (**a**) For OC patients in the entire cohort (*n* = 97), the association had a marginal statistically significant result (GG/GT vs. TT; log-rank test, *p* = 0.054); (**b**) For OC patients in the sub-cohort (*n* = 88), excluding those with prior VTE, TT genotype carriers had a mean PFS of 24.4 ± 3.2 months while G allele carriers had a mean PFS of 43.7 ± 8.1 months (GG/GT vs. TT; log-rank test, *p* = 0.038).

To eliminate potential confounding effects of pre-existing VTE on the relationship between SNPs and patient prognosis, the analyses were performed considering the entire cohort A (*n* = 98) and the subgroup of patients excluding those who had VTE before OC diagnosis (*n* = 89). In this survival analysis, only the *SELP* SNP showed a significant association with the PFS or OS of the patients. The remaining negative results are detailed in Supplementary Table S1. Considering the entire Cohort A, the *SELP* rs6136 TT genotype showed a marginal association with PFS (GG/GT vs. TT; log-rank test, *p* = 0.054; Figure 1a) while being significantly associated with poorer PFS compared to the G allele genotypes (CG/GT vs. TT; mean OS of 24.4 ± 3.2 months and 43.7 ± 8.1 months, respectively, log-rank test, *p* = 0.038; Figure 1b) in subgroup assessment. Despite the statistical significance, it is important to note the underrepresentation of the G allele genotype, which limits the robustness of these results.

### 3.5. Genes’ Impact on Clinical Outcome of OC Patients (Independently of VTE)

**Figure 2:**
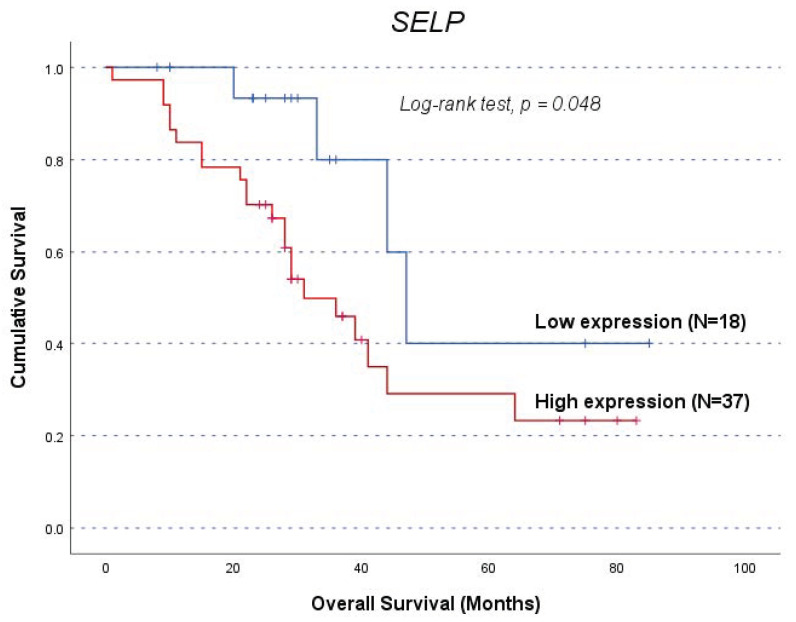
Overall survival (OS) (*n* = 55) by Kaplan−Meier and log-rank test for OC patients, according to *SELP* expression levels. Patients with high *SELP* expression (profile D) had a lower OS compared to those with low *SELP* expression (*p* = 0.048). The high expression group had a mean OS of 41.2 ± 5.1 months, while the low expression group had a mean OS of 57.9 ± 9.2 months.

**Figure 3:**
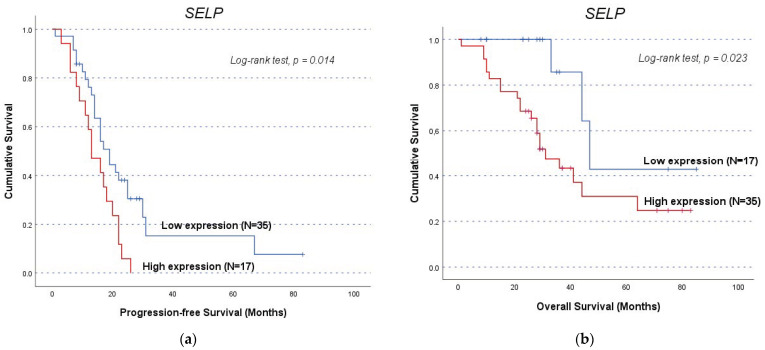
Progression-free survival (PFS) and overall survival (OS) by Kaplan−Meier and log-rank test for OC patients in the sub-cohort (*n* = 52), excluding those with prior VTE, according to *SELP* expression levels: (**a**) Patients with high *SELP* expression had a lower PFS compared to those with low *SELP* expression (profile C). The high expression group had a mean PFS of 14.4 ± 1.7 months, while the low expression group had a mean PFS of 26.3 ± 4.8 months (log-rank test, *p* = 0.014); (**b**) Patients with high *SELP* expression had a lower OS compared to those with low *SELP* expression (profile D). The high expression group had a mean OS of 41.5 ± 5.3 months, while the low expression group had a mean OS of 60.6 ± 9.5 months (log-rank test, *p* = 0.023).

A similar approach to cohort A was used to segment cohort B patients, resulting in a subgroup excluding patients who experienced VTE before OC diagnosis (*n* = 52). In the total cohort B (*n* = 55), patients with high *SELP* expression (profile D) exhibited a shorter OS compared to their counterparts (mean OS of 41.2 ± 5.1 and 57.9 ± 9.2 months, respectively, log-rank test, *p* = 0.048; Figure 2). In the sub-analysis, *SELP* expression not only continued to show a significant association with OS but also displayed an association with PFS (Figure 3). For PFS, the high *SELP* expression (profile C) group had a mean PFS of 14.4 ± 1.7 months compared to 26.3 ± 4.8 months in the low expression group (log-rank test, *p* = 0.014, Figure 3a). Concerning OS, patients with a high expression (profile D) had a lower survival time than their counterparts (mean OS of 41.5 ± 5.3 months and 60.6 ± 9.5 months, log-rank test, *p* = 0.023, Figure 3b). Consistent with the SNP analyses, only *SELP* showed a significant association with OC patient prognosis, further supporting its potential role as a prognostic factor in these patients, with high expression levels associated with a less favourable outcome. The negative findings from all gene analyses conducted are summarised in Supplementary Table S2.

### 3.6. SNPs’ Impact on Gene Expression

Regarding the influence of the SNPs on their respective gene expression levels in cohort B, no significant association was found (Table 4). Considering all cohort B patients (*n* = 55), the *NOS3* rs2070744 T allele (TT/CT vs. CC) was significantly linked with higher levels of *ICAM1* (profile D, χ^2^, *p* = 0.045). The TT genotype showed a marginal association with higher *ENG* expression (CC/CT vs. TT; profile B, χ^2^, *p* = 0.058). Moreover, the *VWF* rs1063856 TT genotype (additive model) was significantly linked to higher *SELP* expression (profile B, χ^2^, *p* = 0.025, respectively). Regarding the subgroup (*n* = 52), the *NOS3* rs2070744 T allele (TT/CT vs. CC) was again associated with higher *ICAM1* expression levels (profile D, χ^2^, *p* = 0.019). The mentioned associations are resumed in Table 5.

## 4. Discussion

Despite advancements in the treatment setting, long-term survival rates for OC remain low due to several challenges, including late-stage disease diagnosis and the frequent development of resistance to therapy allied to the high recurrence rates. Identifying effective prognostic biomarkers of OC is crucial for improving clinical management and personalising therapeutic strategies [1,2,3,6,37]. A growing body of evidence has underscored the crucial role of ED in the initiation and progression of cancer, with its influence extending to various hallmarks of the disease. In parallel, ED has long been recognised as one of the primary factors linked to VTE, a condition commonly observed in cancer patients [36]. The interaction between ED, VTE, and OC could enable the identification of novel biomarkers and therapeutic targets for more personalised disease management for OC patients. This study assessed the association of the ED markers with VTE occurrence and their prognostic value for OC.

Starting with *NOS3* rs2070744, it involves the substitution of a thymine (T) with a cytosine (C) at position -786 in the 5′-flanking region. This alternation promotes the binding of the DNA repair, replication and recombination protein, replication protein A1 (RPA1), to the *NOS3* promoter. This assembly reduces the promoter activity leading to serum NO decline, which enables proliferation pathways and inhibits tumour cell apoptosis [30,38,39,40,41]. In this study, the rs2070744 C allele was more prevalent among patients with a history of CVD and metabolic diseases (CC vs. CT vs. TT; χ^2^, *p =* 0.049), which is consistent with the literature [39,42,43]. Additionally, the SNP was marginally associated with the baseline INR (C vs. TT; χ^2^, *p =* 0.059), corroborating the effect of the SNP on haemostatic abnormalities [44]. Contrary to the observation that decreased NO levels—which define ED status—are associated with a pro-thrombotic potential, rs2070744 was not significantly associated with CAT susceptibility [21]. On the other hand, higher *NOS3* expression was significantly linked to a lower risk of VTE (χ^2^, *p =* 0.013), which aligns with the protective role of NO in maintaining endothelial integrity and preventing excessive clot formation [22,28,45]. Notably, the expression of the remaining genes was not significantly associated with CAT occurrence. Collectively, these results suggest that *NOS3* may have a unique and context-dependent role in CAT pathogenesis among OC patients.

The lack of association between rs2070744 and *NOS3* expression may help explain why this SNP does not appear to influence CAT susceptibility. This finding should be examined in additional studies with larger cohort sizes. Inclusively, while with no statistical significance, the C allele tended to be more common among those with low *NOS3* expression, supporting the existing evidence [30,38,39,40,41]. All in all, the interplay between NO deficiency and the activation of vasoconstrictors exacerbates ED’s role in promoting a pro-thrombotic and pro-inflammatory environment, leading to enhanced cellular proliferation, angiogenesis, and metastasis—key processes in tumourigenesis. This connection further underscores the significance of ED beyond its involvement in VTE, highlighting its contribution to a wider array of pathological conditions that share common mechanistic pathways with cancer [21,22,28,36,39,46,47,48].

The influence of *NOS3* expression on OC may extend to treatment, particularly in the context of PARPi. The association of the rs2070744 TT genotype with higher levels of NO compared to the C allele may reflect a preserved capacity for regulating inflammatory and apoptotic processes. As an apoptosis modulator, elevated NO levels enhance apoptosis in cancer cells, which could potentially impact the effectiveness of PARPi [30,47,49]. The significant association between the TT genotype and the use of PARPi (C vs. TT; χ^2^, *p* = 0.025) suggest that this genotype is linked to a more robust apoptotic response to the treatment, possibly indicating a more favourable clinical outcome or a preferred therapeutic response in OC patients [50]. Another hypothesis suggests that NO may play a role in sensitising *BRCA1*/*2*-proficient tumours to PARPi by inhibiting homologous recombination repair (HRR) pathways. Briefly, *BRCA*-mutated tumours have a reduced ability to repair DNA via HRR, making them more vulnerable to treatments that further inhibit DNA repair, like PARPi. However, *BRCA1*/*2* mutations are only present in 10.0–15.0% of OC, meaning that for most OC patients with *BRCA1*/*2*-proficient tumours, PARPi is less effective. Recently, NO donors have been proposed as such efficient sensitising agents [51]. Thus, the elevated NO levels associated with the TT genotype could enhance the synthetic lethality of PARPi, even in patients without *BRCA1*/*2* mutations, by promoting error-prone DNA repair mechanisms.

Intriguingly, the rs2070744 T allele was associated with higher expression levels of *ICAM1* [χ^2^, *p =* 0.045 (*n* = 55); χ^2^, *p =* 0.019 (*n* = 52)], and marginally associated with the upregulation of *ENG* [χ^2^, *p =* 0.058 (*n* = 55)]. The elevated expression of *ICAM1*, a crucial marker for leukocyte adhesion and endothelial inflammation, suggests a potential exacerbation of inflammatory responses in T allele carriers [52]. Moreover, the increased expression of *ENG*, which plays a key role in EC functions, particularly in regulating proliferation, migration and angiogenesis, could indicate an altered endothelial state with potential impacts on tumour progression [53]. These findings seem to contrast with the expectation that higher NO levels associated with the T allele should mitigate ED and thrombotic risk. Instead, the presence of the T allele appears to be associated with a molecular profile suggesting persistent ED, despite elevated NO levels. Nevertheless, besides being a novel ED marker highly expressed in activated ECs, *ENG* also regulates *NOS3* expression, playing a crucial role in NO-mediated vasodilation, through the TGFβ/activin receptor-like kinase-5 (ALK5)/mothers against decapentaplegic homolog 2 (SMAD2) pathway [26,54]. Thus, the simultaneous upregulation of *ENG* and *NOS3* (assuming the *NOS3* rs2070744 T allele association with higher *NOS3* expression) acts in line with a more preserved endothelium resultant from a regular NO bioavailability, reinforcing the vascular dynamics modulated by *ENG* [30,40,41,54]. Again, the lack of statistical significance on the association of T allele carriers and higher *NOS3* expression (regardless of the observed trend) may be a justifying factor for the mere marginally significant association of T allele carriers with higher *ENG* expression, reflecting a complex interaction between NO levels and other ED markers.

The SNP rs2070744 has been linked to the susceptibility of several tumours, including breast, prostate, bladder, gastric, and colorectal cancers, and oral squamous cell carcinoma [30,36,41,48,55,56,57,58,59,60,61,62]. However, studies reporting a clear prognostic role of rs2070744 in cancer are limited, focusing primarily on its association with survival in response to specific therapies in renal cell carcinoma, hepatocellular carcinoma, and bladder cancer [63,64,65]. Notably, these studies yield inconsistent results regarding which allele or genotype was associated with a worse prognosis. In this study, no prognostic value of rs2070744 or *NOS3* expression was observed.

Regarding the *SELP* rs6136, this SNP is a missense variant resulting from a thymine (T) to guanine (G) substitution and consequent threonine (Thr) replacement by proline (Pro) at position 715, at exon 13. Adjacent to this exon, which encodes the last repeat segment of *SELP*, is the exon encoding the transmembrane domain of the protein. Under alternative splicing, a soluble SELP (sSELP) is produced, without a transmembrane domain. The reported association of the G allele with reduced sSELP levels suggests that this allele is linked to impaired splicing efficiency or altered protein processing, leading to decreased production or increased degradation of sSELP compared to the T allele [66,67].

In the present study, no statistical significance was achieved for *SELP* rs6136 and the expression of the respective gene. Nevertheless, the rs6136 G allele was significantly associated with a CVD and/or metabolic disease history (GG/GT vs. TT; χ^2^, *p =* 0.018), aligning with the literature findings, that link this allele with both higher risk of development of metabolic and CVD (including CAT) and lower sSELP levels [20,66,68,69]. The apparent inconsistency in the literature regarding the simultaneous association of the rs6136 G allele with these two factors remains unresolved. On one side, SELP is reported to facilitate leukocyte recruitment to the site of inflammation, potentially contributing to thrombosis, tumour progression and cancer cachexia [70]. Conversely, studies such as the *Etude Cas-Témoin de l’Infarctus du Myocarde* (ECTIM) have shown a protective effect of the G allele against myocardial infarction, proposing *SELP* rs6136 as a polymorphic variant with a population-dependent role. Furthermore, the alteration in the processing of the mRNA or the protein itself may be favouring the transmembrane form of SELP over sSELP as the reduction of the latter related to the increase of the former [71].

The *SELP* rs6136 T allele displayed a greater prevalence in patients with advanced FIGO stages (χ^2^, *p =* 0.042), indicating a worse prognosis associated with *SELP* upregulation. This finding was further confirmed with the observation that TT genotype carriers in this study exhibited lower PFS in the analysis of the entire cohort A (with a marginal result) and the subgroup analysis without patients that had VTE preceding their OC diagnosis (log-rank test, *p* = 0.038) than patients with the G allele. Consistently, patients with higher levels of *SELP* presented a shorter OS compared to their counterparts in the total cohort B (log-rank test, *p* = 0.048), and both diminished PFS and OS (log-rank test, *p* = 0.014 and *p* = 0.023, respectively) in the sub-analysis cohort dismissing patients with VTE before OC diagnosis. This result aligns with previous studies regarding advanced EOC prognosis, where increased expression of *SELP* mRNA was linked to worse OS [72]. Taken together, this study’s findings suggest that ED and *SELP*’s roles are not merely associated with the presence of VTE but are closely linked with their impact in the context of ongoing cancer [20]. However, this SNP should be evaluated carefully given the underrepresentation of the GG genotype in the study sample, which makes it challenging to draw definitive conclusions about its impact on cancer outcomes. Looking forward, expanding the cohort could enable wider genotype representation and consequently provide better statistical power. Also, combining the quantification of protein levels with gene expression analyses could help clarify these study’s findings, such as sSELP’s role in the complex VTE−ED−OC triad.

As for *VWF*, the variant rs1063856 involves a thymine (T) to cytosine (C) substitution in exon 18, which leads to increased plasma levels of vWF and, consequently a higher thrombosis risk [73,74]. Elevated vWF levels are known to contribute to cancer progression, as vWF can enhance platelet adhesion and aggregation, creating a pro-thrombotic environment that favours tumour metastasis [75]. Curiously, the T allele was significantly associated with a history of ATE in this study (χ^2^, *p =* 0.018). Although controversial, this result aligns with the marginal association of the SNP TT genotype with the use of anticoagulants before their OC diagnosis, suggesting a pro-thrombotic role for this genotype. Although no association was detected between the SNP and the expression level of *VWF* in OC patients’ PBCs, the TT genotype was found to be significantly associated with a higher expression of *SELP* (χ^2^, *p =* 0.025). This finding is consistent with the SNP’s association with ATE and the use of anticoagulants before OC diagnosis. Overall, *VWF* rs1063856 seems to have a role in thrombo-inflammatory mechanisms as SELP is known to mediate the interaction between activated ECs, platelets, and immune cells, a key process in thrombogenesis [16]. Although previous investigations report the association of *VWF* rs1063856 with both vWF plasma levels and VTE incidence, the present study lacks statistical significance for both analyses [74]. Notably, there is a lack of literature assessing the prognostic value of this SNP. In this study, no prognostic value was observed for *VWF* rs1063856, which aligns with the current gap in research regarding the role of this SNP in predicting cancer progression or patient outcomes.

Future research should aim to externally validate these findings in larger cohorts and other populations and perform functional studies to better understand the molecular mechanisms underpinning the associations between ED-related markers and ovarian tumourigenesis. Additionally, it will be important to conduct active screenings of CAT and integrate the flow-mediated dilation (FMD) test into future studies to directly measure and confirm the presence of ED in OC patients. Finally, in addition to PBCs, the expression of ED-related genes should be evaluated in endothelial cells—the primary source—to gain a better understanding of the gene expression dynamics.

## 5. Conclusions

Over the last few years, the search for reliable prognostic biomarkers has become a priority to improve OC management and patient outcomes. In the cancer research field, markers related to ED have garnered significant interest due to their roles in tumour invasion, angiogenesis and metastasis. Given the dual role of ED in CAT and cancer progression (independently of VTE), the present study aimed to assess the impact of specific ED-related genetic variations and genes on OC-related VTE occurrence, OC progression, and patient survival. Among the evaluated markers, only *NOS3* expression was significantly associated with VTE occurrence. Namely, its higher expression was linked to a reduced risk of VTE, reinforcing the idea of a protective effect of NO in venous thrombogenesis. This is attributed, at least partially, to the maintenance of endothelial integrity and normal activity, a well-established role of NO in cardiovascular research. Another important finding relates to SELP*,* a crucial mediator of endothelial integrity and tumour angiogenesis. In this study, *SELP* expression was associated with poor clinical outcomes. As for the SNP, despite demonstrating a strong correlation with poorer survival, no definitive conclusions may be taken due to limiting underrepresentation of its genotypes. Although more studies with larger cohort sizes and diverse populations are needed, together the findings of this preliminary study suggest that ED-related markers have a significant yet context-dependent role in ovarian tumourigenesis, which goes beyond venous thrombogenesis. Thus, additional research could pave the way for the identification of novel prognostic biomarkers for OC management, which are currently needed to improve patient clinical outcomes. Since one biomarker is most likely to be insufficient to significantly improve the accuracy of prognosis assessment, the goal should be to identify and combine multiple ED-related markers with a prognostic value in OC into a profile. Indeed, future studies should investigate a broader spectrum of ED-related genes to aid in the development of targeted therapies that address both cancer progression and thrombosis, offering new avenues for personalised treatment of OC.

## Figures and Tables

**Table 1:** Genotype distribution of ED-related SNPs.

SNP	MAFi ^1^ (MA)	Genotype	*n* (%)	*n* Total (%)	MAFs (MA)
*NOS3* rs2070744	49.5% (C)	CC	21 (21.4)	98 (100)	46.9% (C)
		CT	50 (51.0)		
		TT	27 (27.6)		
*SELP* rs6136	8.9% (G)	TT	81 (82.7)	98 (100)	9.2% (G)
		GT	16 (16.3)		
		GG	1 (1.0)		
*VWF* rs1063856	34.1% (C)	CC	10 (10.2)	98 (100)	36.2% (C)
		CT	51 (52.0)		
		TT	37 (37.8)		

^1^ According to the Ensembl database accessed on 18 August 2024. Abbreviations: SNP, single-nucleotide polymorphism; MAFi, minor allele frequency in the Iberian population; MAFs, minor allele frequency in the study cohort; MA, minor allele.

**Table 2:** Associations between SNPs and clinicopathological features of the patients.

SNP	Associated Characteristic	Associated Genotype/Allele	Statistical Model	*p* Value
*NOS3* rs2070744	Cardiovascular or metabolic disease history	CC	CC vs. CT vs. TT	0.049
	Cardiovascular and metabolic disease history	CC	CC vs. CT/TT	0.023
	Use of PARPi	TT	CC vs. CT vs. TT	0.029
		TT	CC/CT vs. TT	0.025
	Higher INR	TT	CC/CT vs. TT	0.059
*SELP* rs6136	Cardiovascular and/or metabolic disease history	G	GG/GT vs. TT	0.018
	Advanced (III/IV) FIGO stages	TT	GG vs. GT vs. TT	0.042
*VWF* rs1063856	ATE history	TT	CC/CT vs. TT	0.018
		TT	CC vs. CT vs. TT	0.021
	Anticoagulant use before OC diagnosis	TT	CC vs. CT vs. TT	0.054

Abbreviations: SNP, single-nucleotide polymorphism; PARPi, poly (ADP-ribose) polymerase (PARP) inhibitors; INR, international normalised ratio; ATE, arterial thromboembolism.

**Table 3:** Significant associations of *NOS3* expression and VTE occurrence.

Gene	Profile	*n*		Expression		*p* Value
			Low	Intermediate	High	
*NOS3*	B	VTE negative	10	12	18	0.013
		VTE positive	5	4	0	
	B	VTE-free	10	12	17	0.027
		VTE before OC	2	1	0	
		VTE after OC	3	3	0	

Abbreviations: VTE, venous thromboembolism; OC, ovarian cancer.

**Table 4:** Genotype distribution of each SNP (additive model) in cohort B (*n* = 55), according to the expression profile A of the respective gene.

SNP	Genotype	Low Gene Expression *n* (%)	High Gene Expression *n* (%)
*NOS3* rs2070744	CC	8 (14.5)	6 (10.9)
	CT	15 (27.3)	12 (21.8)
	TT	4 (7.3)	10 (18.2)
*SELP* rs6136	TT	22 (40.0)	23 (41.8)
	GT	5 (9.1)	5 (9.1)

Abbreviations: SNP, single-nucleotide polymorphism.

**Table 5:** Significant associations between investigated SNPs and gene expression.

SNP	Genotype/ Allele	Statistical Model	Upregulated Gene	Gene Expression Profile	*p* Value	Cohort
*NOS3* rs2070744	T	TT/CT vs. CC	*ICAM1*	D	0.019	Sub-cohort (*n* = 52)
	TT	CC/CT vs. TT	*ENG*	B	0.058	Entire cohort B (*n* = 55)
	T	TT/CT vs. CC	*ICAM1*	D	0.045	
*VWF* rs1063856	TT	CC vs. CT vs. TT	*SELP*	B	0.025	

Abbreviations: SNP, single-nucleotide polymorphism.

## Data Availability

The data presented in this study are available on request from the corresponding author.

## Supplementary Materials

The following supporting information can be downloaded at: https://www.mdpi.com/article/10.3390/life14121630/s1, Supplementary Table S1. Negative results on the associations between investigated SNPs and patient survival. Supplementary Table S2. Negative results on the associations between the investigated genes and patient survival.
