# Supplementary material for: Endothelial Dysfunction Markers in Ovarian Cancer: VTE Risk and Tumour Prognostic Outcomes
Source: Life (Basel). 2024 Dec 9;14(12):1630. doi: 10.3390/life14121630 (PMC11678387; doi:10.3390/life14121630)
Supplement: Supplementary file 1 [file life-14-01630-s001.zip › life-3322051-supplementary.pdf]

Supplementary Table S1. Negative results on the associations between investigated SNPs and patient survival.

SNP	Model		N total	N event	Censured (%)	ST	Mean ST	HR (95% CI)	*P* value
**Cohort A (N=98)**									
NOS3 rs2070744	CC/CT vs. TT [1]	TT	26	21	5 (19.2)	PFS	25.9	4.7 (16.8 – 35.1)	0.679
		C	71	47	24 (33.8)		28.6	3.9 (20.9 – 36.3)	
	TT/CT vs. CC [1]	CC	21	13	8 (38.1)		35.0	7.7 (19.9 – 50.0)	0.435
		T	76	55	21 (27.6)		25.8	3.1 (19.7 – 31.9)	
	CC/CT vs. TT	TT	27	17	10 (37.0)	OS	41.8	5.7 (30.7 – 52.9)	0.338
		C	71	28	43 (60.6)		48.8	4.6 (39.7 – 57.9)	
	TT/CT vs. CC	CC	21	6	15 (71.4)		59.6	7.6 (44.6 – 74.5)	0.163
		T	77	39	38 (49.4)		43.9	3.9 (36.2 – 51.6)	
SELP rs6136 [2]	GG/GT vs. TT	TT	81	38	43 (53.1)	OS	45.6	4.0 (37.7 – 53.4)	0.484
		G	17	7	10 (58.8)		48.9	7.5 (34.3 – 63.5)	
VWF rs1063856	CC/CT vs. TT [1]	TT	37	23	14 (37.8)	PFS	29.5	5.7 (18.3 -40.6)	0.626
		C	60	45	15 (25.0)		26.5	3.6 (19.4 – 33.6)	
	TT/CT vs. CC [1]	CC	10	7	3 (30.0)		14.6	2.4 (10.0-19.3)	0.190
		T	87	61	26 (29.9)		28.5	3.3 (22.1 – 34.5)	
	CC/CT vs. TT	TT	37	17	20 (54.1)	OS	43.3	6.1 (31.3 – 55.3)	0.504
		C	61	28	33 (54.1)		48.6	4.5 (39.8 – 57.4)	
	TT/CT vs. CC	CC	10	6	4 (40.0)		32.3	4.5 (23.6 – 41.0)	0.241
		T	88	39	49 (55.7)		48.5	3.9 (40.9 – 56.2)	
**Sub-cohort excluding patients with VTE before OC diagnosis (N=89)**									
NOS3 rs2070744	CC/CT vs. TT [1]	TT	24	19	5 (20.8)	PFS	27.1	5.0 (17.3 - 36.9)	0.839
		C	64	42	22 (34.4)		28.9	4.2 (20.6 – 37.1)	
	TT/CT vs. CC [1]	CC	19	12	7 (36.8)		32.5	8.2 (16.5 – 48.5)	0.659
		T	69	49	20 (29.0)		27.0	3.4 (20.3 – 33.7)	
	CC/CT vs. TT	TT	25	15	10 (40.0)	OS	44.0	5.9 (32.3 – 55.6)	0.392
		C	64	24	40 (62.5)		50.7	4.9 (41.1 – 60.3)	
	TT/CT vs. CC	CC	19	5	14 (73.7)		60.8	8.0 (45.1 – 76.6)	0.182
		T	70	34	36 (51.4)		45.7	4.2 (37.5 – 53.9)	
SELP rs6136 [2]	GG/GT vs. TT	TT	73	32	41 (56.2)	OS	47.9	4.3 (39.5 – 56.2)	0.729
		G	16	7	9 (56.3)		48.2	7.5 (33.4 – 62.9)	
VWF rs1063856	CC/CT vs. TT [1]	TT	31	19	12 (38.7)	PFS	29.9	6.1 (17.9 – 41.8)	0.582
		C	57	42	15 (26.3)		27.3	3.8 (19.8 – 34.9)	
	TT/CT vs. CC [1]	CC	10	7	3 (30.0)		14.6	2.4 (10.0 – 19.3)	0.184
		T	78	54	24 (30.8)		29.3	3.5 (22.3 – 36.2)	
	CC/CT vs. TT	TT	31	13	18 (58.1)	OS	45.9	6.6 (32.8 – 58.9)	0.819
		C	58	26	32 (55.2)		49.5	4.6 (40.5 – 58.6)	
	TT/CT vs. CC	CC	10	6	4 (40.0)		32.3	4.6 (23.6 – 41.0)	0.154
		T	79	33	46 (58.2)		50.7	4.1 (42.6 – 58.8)	

[1] The sum of absolute values may not equal the total cohort size due to missing data for some variables. [2] No statistical tests were computed for the TT/GT vs. GG model as all cases were censored. Abbreviations: SNP, single-nucleotide polymorphism; ST, survival time; OS, overall survival; PFS, progression-free survival; HR, hazard ration; CI, confidence interval.

Supplementary Table S2. Negative results on the associations between the investigated genes and patient survival.

Gene	Profile[1]	Expression	N total	N event	Censured (%)	ST	Mean ST	HR (95% CI)	*P* value
**Cohort B (N=55)**									
NOS3	A	Low	27	21	6 (22.2)	PFS	22.3	5.0 (12.5 – 32.2)	0.903
		High	28	24	4 (14.3)		21.0	3.3 (14.5 – 27.5)	
	B	Low	18	15	3 (16.7)		16.6	2.3 (12.1 – 21.1)	0.279
		Intermediate	19	13	6 (31.6)		29.2	6.8 (15.9 – 42.5)	
		High	18	17	1 (5.6)		17.3	1.6 (14.1 – 20.5)	
	C	Low	37	28	9 (24.3)		24.0	4.3 (15.5 – 32.5)	0.336
		High	18	17	1 (5.6)		17.3	1.6 (14.1 – 20.5)	
	D	Low	18	15	3 (16.7)		16.6	2.3 (12.1 – 21.1)	0.498
		High	37	30	7 (18.9)		22.9	3.6 (15.9 – 30.0)	
	A	Low	27	11	16 (59.3)	OS	48.6	7.2 (34.6 – 62.7)	0.512
		High	28	15	13 (46.4)		42.3	5.5 (31.5 – 53.1)	
	B	Low	18	9	9 (50.0)		43.1	8.3 (26.8 – 59.5)	0.869
		Intermediate	19	8	11 (57.9)		46.7	8.1 (30.9 – 62.6)	
		High	18	9	9 (50.0)		44.3	6.0 (32.4 – 56.1)	
	C	Low	37	17	20 (54.1)		45.2	5.9 (33.5 – 56.8)	0.823
		High	18	9	9 (50.0)		44.3	6.0 (32.4 – 56.1)	
	D	Low	18	9	9 (50.0)		43.1	8.3 (26.8 – 59.5)	0.598
		High	37	17	20 (54.1)		47.4	5.4 (36.8 – 58.0)	
SELP	A	Low	27	21	6 (22.2)	PFS	20.8	4.0 (12.9 – 28.7)	0.667
		High	28	24	4 (14.3)		22.0.	4.1 (13.9 – 30.1)	
	B	Low	18	12	6 (33.3)		19.5	2.1 (15.4 – 23.6)	0.182
		Intermediate	19	15	4 (21.1)		28.1	6.5 (15.2 – 40.9)	
		High	18	18	0 (0.0)		15.4	1.8 (11.8 – 19.0)	
	C	Low	37	27	10 (27.0)		25.5	4.5 (16.6 – 34.3)	0.070
		High	18	18	0 (0.0)		15.4	1.8 (11.8 – 19.0)	
	D	Low	18	12	6 (33.3)		19.5	2.1 (15.4 – 23.6)	0.637
		High	37	33	4 (10.8)		21.3	3.5 (14.6 – 28.1)	
	A	Low	27	10	17 (63.0)	OS	50.0	7.7 (34.8 – 65.1)	0.550
		High	28	16	12 (42.9)		43.8	5.6 (32.9 – 54.7)	
	B	Low	18	4	14 (77.8)		57.9	9.2 (39.8 – 76.0)	0.142
		Intermediate	19	10	9 (47.4)		47.0	7.7 (31.9 – 62.2)	
		High	18	12	6 (33.3)		38.5	5.3 (28.1 – 48.8)	
	C	Low	37	14	23 (62.2)		51.9	6.3 (39.6 – 64.3)	0.285
		High	18	12	6 (33.3)		38.5	5.3 (28.1 – 48.8)	
ICAM1	A	Low	27	22	5 (18.5)	PFS	22.0	3.9 (14.3 – 29.7)	0.728
		High	28	23	5 (17.9)		21.1	4.2 (12.8 – 29.2)	
	B	Low	18	16	2 (11.1)		21.6	4.1 (13.4 – 29.7)	0.950
		Intermediate	19	13	6 (31.6)		16.5	2.0 (12.6 – 20.4)	
		High	18	16	2 (11.1)		21.8	4.5 (12.9 – 30.7)	
	C	Low	37	29	8 (21.6)		21.3	3.8 (13.7 – 28.8)	0.931
		High	18	16	2 (11.1)		21.8	4.5 (12.9 – 30.7)	
	D	Low	18	16	2 (11.1)		21.6	4.1 (13.4 – 29.7)	0.843
		High	37	29	8 (21.6)		21.4	3.9 (13.7 – 29.2)	
	A	Low	27	14	13 (48.1)	OS	47.3	5.9 (35.7 – 58.8)	0.669
		High	28	12	16 (57.1)		47.9	6.4 (35.4 – 60.4)	
	B	Low	18	9	9 (50.0)		51.1	7.3 (36.8 – 65.4)	0.607
		Intermediate	19	8	11 (57.9)		40.8	5.7 (29.6 – 52.0)	
		High	18	9	9 (50.0)		47.7	7.6 (32.9 – 62.6)	
	C	Low	37	17	20 (54.1)		47.8	5.4 (37.2 – 58.4)	0.418
		High	18	9	9 (50.0)		47.7	7.6 (32.9 – 62.6)	

	D	Low	18	9	9 (50.0)		51.1	7.3 (36.8 – 65.4)	0.357
		High	37	17	20 (54.1)		41.2	5.3 (30.7 – 51.7)	
ENG	A	Low	27	21	6 (22.2)	PFS	18.7	1.8 (15.1 – 22.2)	0.971
		High	28	24	4 (14.3)		25.2	4.6 (16.1 – 34.3)	
	B	Low	18	15	3 (16.7)		18.1	2.3 (13.6 – 22.6)	0.656
		Intermediate	9	13	6 (31.6)		26.7	7.4 (12.1 – 41.3)	
		High	18	17	1 (5.6)		20.9	4.2 (12.7 – 29.2)	
	C	Low	37	28	9 (24.3)		22.0	3.9 (14.7 – 29.6)	0.476
		High	18	17	1 (5.6)		20.9	4.2 (12.7 – 29.2)	
	D	Low	18	15	3 (16.7)		18.1	2.3 (13.6 – 22.6)	0.867
		High	37	30	7 (18.9)		23.5	4.0 (15.6 – 31.4)	
	A	Low	27	11	16 (59.3)	OS	47.3	6.7 (34.1 – 60.5)	0.519
		High	28	15	13 (46.4)		46.0	6.2 (33.9 – 58.1)	
	B	Low	18	7	11 (61.1)		47.9	8.6 (31.2 – 64.7)	0.498
		Intermediate	19	9	10 (52.6)		47.5	6.8 (34.2 – 60.8)	
		High	18	10	8 (44.4)		45.6	7.2 (31.4 – 59.8)	
	C	Low	37	16	21 (56.8)		47.8	5.4 (31.4 – 59.8)	0.239
		High	18	10	8 (44.4)		45.6	7.2 (31.4 – 59.8)	
	D	Low	18	7	11 (61.1)		47.9	8.6 (31.2 – 64.7)	0.563
		High	37	19	18 (48.6)		45.1	5.4 (34.6 – 55.6)	
EDN1	A	Low	27	20	7 (25.9)	PFS	17.3	1.2 (14.2 – 20.3)	0.680
		High	28	25	3 (10.7)		22.5	3.9 (14.9 – 30.1)	
	B	Low	18	12	6 (33.3)		16.8	2.3 (12.4 – 21.2)	0.656
		Intermediate	19	16	3 (15.8)		20.5	1.7 (17.3 – 23.8)	
		High	18	17	1 (5.6)		20.8	4.8 (11.4 – 30.3)	
	C	Low	37	28	9 (24.3)		18.9	1.5 (16.0 – 21.7)	0.513
		High	18	17	1 (5.6)		20.8	4.8 (11.4-30.3)	
	D	Low	18	12	6 (33.3)		16.8	2.3 (12.4 – 21.2)	0.728
		High	37	33	4 (10.8)		22.1	3.2 (15.8 – 28.3)	
	A	Low	27	10	17 (63.0)	OS	44.0	7.3 (29.7 – 58.3)	0.862
		High	28	16	12 (42.9)		45.7	5.4 (35.1 – 56.2)	
	B	Low	18	5	13 (72.2)		55.6	7.3 (41.3 – 69.9)	0.751
		Intermediate	19	8	11 (57.9)		42.4	6.5 (29.7 – 55.1)	
		High	18	13	5 (27.8)		42.7	6.2 (30.5 – 54.9)	
	C	Low	37	13	24 (64.9)		45.5	5.9 (33.9 - 57.1)	0.451
		High	18	13	5 (27.8)		42.7	6.2 (30.5 – 54.9)	
	D	Low	18	5	13 (72.2)		55.6	7.3 (41.3 – 69.9)	0.774
		High	37	21	16 (43.2)		45.1	4.8 (35.6 – 54.5)	
**Sub-cohort excluding patients with VTE before OC diagnosis (N=52)**									
NOS3	A	Low	25	19	6 (24.0)	PFS	23.2	5.4 (12.6 – 33.9)	0.770
		High	27	23	4 (14.8)		21.1	3.8 (13.7 – 28.6)	
	B	Low	16	13	3 (18.8)		17.1	2.3 (1.1 – 22.1)	0.485
		Intermediate	18	12	6 (33.3)		31.0	7.3 (16.7 – 45.2)	
		High	18	17	1 (5.6)		17.3	1.6 (14.1 – 20.5)	
	C	Low	34	25	9 (26.5)		25.7	5.0 (15.9 – 35.5)	0.340
		High	18	17	1 (5.6)		17.3	1.6 (14.1 – 20.5)	
	D	Low	16	13	3 (18.8)		17.1	2.3 (1.1 – 22.1)	0.857
		High	36	29	7 (19.4)		23.2	3.9 (15.7 – 30.8)	
	A	Low	25	10	15 (60.0)	OS	49.5	7.4 (35.1 – 64.0)	0.468
		High	27	14	13 (48.1)		43.4	5.9 (31.9 – 54.9)	
	B	Low	16	8	8 (50.0)		43.8	8.7 (26.7 – 60.9)	0.892
		Intermediate	18	7	11 (61.1)		48.6	8.8 (31.3 – 65.9)	
		High	18	9	9 (50.0)		44.3	6.0 (32.4 – 56.1)	
	C	Low	34	15	19 (55.9)		46.6	6.4 (34.1 – 59.1)	0.937
		High	18	9	9 (50.0)		44.3	6.0 (32.4 – 56.1)	

	D	Low	16	8	8 (50.0)		43.8	8.7 (26.7 – 60.9)	0.654
		High	36	16	20 (55.6)		48.5	5.6 (37.5 – 59.6)	
SELP	A	Low	25	19	6 (24.0)	PFS	21.6	4.4 (13.1 – 30.2)	0.967
		High	27	23	4 (14.8)		22.5	4.8 (13.1 – 31.9)	
	D	Low	17	11	6 (35.3)		20.0	2.2 (15.7 – 24.3)	0.461
		High	35	31	4 (11.4)		21.9	3.8 (14.4 – 29.4)	
	A	Low	25	9	16 (64.0)	OS	50.9	8.0 (35.3 – 66.5)	0.489
		High	27	15	12 (44.4)		44.6	5.9 (33.2 – 56.1)	
	B	Low	17	3	14 (82.4)		60.6	9.5 (42.0 – 79.2)	0.075
		Intermediate	18	10	8 (44.4)		45.7	7.8 (30.3 – 61.1)	
		High	17	11	6 (35.3)		38.7	5.7 (27.6 – 49.9)	
	C	Low	35	13	22 (62.9)		52.5	6.4 (39.9 – 65.2)	0.262
		High	17	11	6 (35.3)		38.7	5.7 (27.6 – 49.9)	
ICAM1	A	Low	25	20	5 (20.0)	PFS	22.6	4.6 (13.6 – 3.6)	0.865
		High	27	22	5 (18.5)		21.3	4.4 (12.8 – 29.9)	
	B	Low	16	14	2 (12.5)		21.5	4.6 (12.5 – 30.6)	0.944
		Intermediate	19	13	6 (31.6)		16.5	2.0 (12.6 – 20.4)	
		High	17	15	2 (11.8)		22.3	4.8 (13.0 – 31.7)	
	C	Low	35	27	8 (22.9)		22.3	4.6 (13.2 – 31.3)	0.785
		High	17	15	2 (11.8)		22.3	4.8 (13.0 – 31.7)	
	D	Low	16	14	2 (12.5)		21.5	4.6 (12.5 – 30.6)	0.990
		High	36	28	8 (22.2)		21.7	4.0 (13.8 – 29.6)	
	A	Low	25	13	12 (48.0)	OS	47.6	6.2 (35.5 – 59.8)	0.851
		High	27	11	16 (59.3)		49.0	6.5 (36.2 – 61.8)	
	B	Low	16	8	8 (50.0)		51.7	7.9 (36.3 – 67.1)	0.731
		Intermediate	19	8	11 (57.9)		40.8	5.7 (29.6 – 52.0)	
		High	17	8	9 852.9)		49.4	7.8 (34.0 – 64.7)	
	C	Low	35	16	19 (54.3)		48.3	5.6 (37.3 – 59.4)	0.609
		High	17	8	9 852.9)		49.4	7.8 (34.0 – 64.7)	
	D	Low	16	8	8 (50.0)		51.7	7.9 (36.3 – 67.1)	0.435
		High	36	16	20 (55.6)		41.8	5.5 (31.1 – 52.3)	
ENG	A	Low	25	19	6 (24.0)	PFS	18.3	1.8 (14.7 – 2.9)	0.939
		High	27	23	4 (14.8)		25.6	4.9 (16.3 – 35.0)	
	B	Low	16	13	3 (18.8)		17.5	2.3 (12.9 – 22.1)	0.687
		Intermediate	19	13	6 (31.6)		26.7	7.4 (12.1 – 41.3)	
		High	17	16	1 (5.9)		21.1	4.5 (12.7 – 30.1)	
	C	Low	35	26	9 (25.7)		22.6	4.6 (13.7 – 31.6)	0.572
		High	17	16	1 (5.9)		21.1	4.5 (12.7 – 30.1)	
	D	Low	16	13	3 (18.8)		17.5	2.3 (12.9 – 22.1)	0.741
		High	36	29	7 (19.4)		23.8	4.2 (15.7 – 31.9)	
	A	Low	25	10	15 (60.0)	OS	48.2	7.2 (34.0 – 62.3)	0.629
		High	27	14	13 (48.1)		46.9	6.3 (34.6 – 59.3)	
	B	Low	16	6	10 (62.5)		49.3	9.5 (30.6 – 67.9)	0.658
		Intermediate	19	9	10 (52.6)		47.5	6.8 (34.2 – 60.8)	
		High	17	9	8 (47.1)		47.1	7.5 (32.4 – 61.8)	
	C	Low	35	15	20 (57.1)		48.5	5.7 (37.3 – 59.6)	0.366
		High	17	9	8 (47.1)		47.1	7.5 (32.4 – 61.8)	
	D	Low	16	6	10 (62.5)		49.3	9.5 (30.6 – 67.9)	0.615
		High	36	18	18 (50.0)		45.8	5.5 (35.1 – 56.5)	
EDN1	A	Low	26	19	7 (26.9)	PFS	17.5	1.6 (14.3 – 20.6)	0.883
		High	26	23	3 (11.5)		22.8	4.3 (14.4 – 31.2)	
	B	Low	17	11	6 (35.3)		17.3	2.4 (12.7 – 21.9)	0.711
		Intermediate	17	14	3 (17.6)		20.1	1.6 (17.0 – 23.2)	
		High	18	17	1 (5.6)		20.8	4.8 (11.4 – 30.3)	
	C	Low	34	25	9 (26.5)		18.7	1.5 (15.8 – 21.5)	0.461

	High	18	17	1 (5.6)		20.8	4.8 (11.4 – 30.3)	
D	Low	17	11	6 (35.3)		17.3	2.4 (12.7 – 21.9)	0.975
	High	35	31	4 (11.4)		22.4	3.5 (15.5 – 29.2)	
A	Low	26	10	16 (61.5)	OS	43.3	7.3 (29.0 – 57.6)	0.681
	High	26	14	12 (46.2)		47.5	5.8 (36.2 – 58.9)	
B	Low	17	5	12 (70.6)		54.4	7.7 (39.3 – 69.4)	0.534
	Intermediate	17	6	11 (64.7)		47.0	8.0 (31.4 – 62.6)	
	High	18	13	5 (27.8)		42.7	6.2 (30.5 – 54.9)	
C	Low	34	11	23 (67.6)		48.5	6.2 (36.3 – 60.8)	0.308
	High	18	13	5 (27.8)		42.7	6.2 (30.5 – 54.9)	
D	Low	17	5	12 (70.6)		54.4	7.7 (39.3 – 69.4)	0.945
	High	35	19	16 (45.7)		46.5	5.1 (36.5 – 56.5)	

[1] Expression profiles were defined categorising gene-normalised relative expression: A) low versus (vs.) high considering median value expression; B) low vs. intermediate vs. high expression values considering the terciles; C) low (first and second terciles) vs. high (third tercile) expression; and D) low (first tercile) vs. high (remaining distribution) by combining the terciles. Abbreviations: ST, survival time; OS, overall survival; PFS, progression-free survival; HR, hazard ration; CI, confidence interval.
